# Supplementary material for: Evaluation of Complexity Measurement Tools for Correlations with Health-Related Outcomes, Health Care Costs and Impacts on Healthcare Providers: A Scoping Review
Source: Int J Environ Res Public Health. 2022 Dec 1;19(23):16113. doi: 10.3390/ijerph192316113 (PMC9741446; doi:10.3390/ijerph192316113)
Supplement: Supplementary file 1 [file ijerph-19-16113-s001.zip › supplementary 1 2.pdf]

International Journal of
Environmental Research and Public Health

MDPI

Supplementary Materials

Supplementary 1: Search strategies:

The following keywords and Boolean operators were used to find studies of interest.

MEDLINE search strategy:

#01. patient complexity.mp.
#02. complex patient.mp.
#03. complicated patient.mp.
#04. complex health need*.mp.
#05. 1 or 2 or 3 or 4
#06. exp Risk Management/
#07. exp Health Status Indicators/
#08. exp Risk Assessment/
#09. exp Risk Factors/
#10. exp Geriatric Assessment/
#11. screening.mp
#12. risk prediction.mp.
#13. tools.mp.
#14. measure*.mp.
#15. score.mp.
#16. index.mp.
#17. 6 or 7 or 8 or 9 or 10 or 11 or 12 or 13 or 14 or 15 or 16
#18. 5 and 17

CINAHL search strategy:

S01 TI “patient complexity” OR AB “patient complexity”
S02 TI "complex patient" OR AB "complex patient"
S03 TI “complicated patient” OR AB “complicated patient”
S04 TI “complex health need*” OR AB “complex health need*”
S05 S1 OR S2 OR S3 OR S4
S06 (MH "Risk Management"+)
S07 (MH “Clinical Assessment Tools” +)
S08 (MH "Risk Assessment"+)
S09 (MH “Risk Factors” +)
S10 (MH "Geriatric Assessment"+)
S11 TI screening OR AB screening
S12 TI “risk prediction” OR AB “risk prediction”
S13 TI tools OR AB tools
S14 TI score OR AB score
S16 TI index OR AB index
S17 S6 OR S7 OR S8 OR S9 OR S10 OR S11 OR S12 OR S13 OR S14 OR S15 OR S16

S18 S5 AND S17

International Journal of Environmental Research and Public Health

MDPI

Supplementary 2: Correlation between the measured complexity and the outcomes

		Complexity measurement tools							
		**IN-TERMED**	**IN-TERMED for El-derly**	**IM-E-SA**	**COMPRI**	**PCAM**	**COM-PLEXedex**	**SWAAT**	**Bandini's tool**
Patient Out-comes	length of hospital stays / Hospital inpatient utilization	5(4) [1-5]			3(3) [2, 5, 6]	2(2) [7, 8]	1(1) [9]	1(1) [10]	1(1) [11]
	Physical health	1(1) [2]		2(1) [12, 13]	1(1) [2]				
	Mental Health	3(3) [2-4]	(1) [14]	3(2)[12, 13, 15]	1(1) [2]				
	the number of medical diagnoses/ comorbidity levels	2(1) [3, 4]		1(1) [15]					
	the number of medications	1(1) [1]							
	Nurse care intervention	1(1) [1]							
	Social care intervention							1(1) [10]	
	Specialist consultation	2(1) [1, 3]							
	Primary Health Care Use / discharge related care	1(1) [4]		1(0) [16]				1(1) [10]	
	Health-related quality of life	1(1) [5]	2(1) [14, 17]	4(2) [13, 15-17]	1(1) [5]	1(1) [8]			
	Life satisfaction			1(1) [15]					

	Personal resources (e.g. Optimism)		1(1) [18]						
	Support / Social resources	1(1) [4]	1(1) [18]	1(0) [12]					
	Nutrition			2(1) [12]					
	Alcohol / substance misuse					1(1) [19]			
	the number of days aging in the place / probability of home discharge		1(0) [17]	1(0) [17]					1(1) [11]
	Activities of daily living	1(1) [20]		1(1) [12]					
	Cognitive decline	1(1) [20]							
	Oral status and oral health			3(3) [21-23]					
Health care costs	total healthcare costs per patient		1(1) [14]	1(1) [24]					
Impacts on health care providers	the burden for health-related staff					1(1) [25]			

Note: 1-5 means the number of included studies. The numbers in brackets (1-5) means the number of studies which showed correlations with the items and patient complexity. [#] means reference number. Interventional studies are **[9, 12, 16, 17]**

References

1. de Jonge, P., et al., *INTERMED - A clinical instrument for biopsychosocial assessment.* Psychosomatics, 2001. **42**(2): p. 106-109.
2. de Jonge, P., et al., *Medical inpatients at risk of extended hospital stay and poor discharge health status: Detection with COMPRI and INTERMED.* Psychosomatic Medicine, 2003. **65**(4): p. 534-541.
3. Lobo, E., et al., *Identification of components of health complexity on internal medicine units by means of the INTERMED method.* International Journal of Clinical Practice, 2015. **69**(11): p. 1377-1386.
4. de Oliveira, C.A., et al., *Health complexity assessment in primary care: A validity and feasibility study of the INTERMED tool.* Plos One, 2022. **17**(2).
5. De Jonge, P., C.H.M. Latour, and F.J. Huyse, *Implementing psychiatric interventions on a medical ward: Effects on patients' quality of life and length of hospital stay.* Psychosomatic Medicine, 2003. **65**(6): p. 997-1002.
6. Yokokawa, D., et al., *Does scoring patient complexity using COMPRI predict the length of hospital stay? A multicentre case-control study in Japan.* BMJ Open, 2022. **12**(4): p. e051891.
7. Yoshida, S., et al., *Validity and reliability of the Patient Centred Assessment Method for patient complexity and relationship with hospital length of stay: a prospective cohort study.* BMJ Open, 2017. **7**(5): p. e016175.
8. Hewner, S., et al., *Integrating Social Determinants of Health into Primary Care Clinical and Informational Workflow during Care Transitions.* EGEMS (Washington, DC), 2017. **5**(2): p. 2-2.
9. Hawner, S., et al., *Aligning population-based care management with chronic disease complexity.* Nursing Outlook, 2014. **62**(4): p. 250-258.
10. Boutin-Foster, C., et al., *Social work admission assessment tool for identifying patients in need of comprehensive social work evaluation.* Health & Social Work, 2005. **30**(2): p. 117-125.
11. Bandini, F., et al., *Complexity in internal medicine wards: A novel screening method and implications for management.* Journal of Evaluation in Clinical Practice, 2018. **24**(1): p. 285-292.
12. Spoorenberg, S.L., et al., *Health-Related Problems and Changes After 1 Year as Assessed With the Geriatric ICF Core Set (GeriatrICS) in Community-Living Older Adults Who Are Frail Receiving Person-Centered and Integrated Care From Embrace.* Archives of Physical Medicine and Rehabilitation, 2019. **100**(12): p. 2334-2345.
13. Dortland, A., et al., *Assessment of Biopsychosocial Complexity and Health Care Needs: Measurement Properties of the INTERMED Self-Assessment Version.* Psychosomatic Medicine, 2017. **79**(4): p. 485-492.
14. Wild, B., et al., *Significance and costs of complex biopsychosocial health care needs in elderly people: results of a population-based study.* Psychosom Med, 2014. **76**(7): p. 497-502.
15. Peters, L.L., et al., *Development and measurement properties of the self assessment version of the INTERMED for the elderly to assess case complexity.* J Psychosom Res, 2013. **74**(6): p. 518-22.
16. Spoorenberg, S.L.W., et al., *Effects of a population-based, person-centred and integrated care service on health, wellbeing and self-management of community-living older adults: A randomised controlled trial on Embrace.* Plos One, 2018. **13**(1).
17. Uittenbroek, R.J., et al., *Integrated and Person-Centered Care for Community-Living Older Adults: A Cost-Effectiveness Study.* Health Services Research, 2018. **53**(5): p. 3471-3494.
18. Boehlen, F.H., et al., *Self-perceived coping resources of middle-aged and older adults - results of a large population-based study.* Aging & Mental Health, 2017. **21**(12): p. 1303-1309.
19. Sugiyama, Y., M. Matsushima, and H. Yoshimoto, *Association between alcohol consumption/alcohol use disorders and patient complexity: a cross-sectional study.* BMJ Open, 2020. **10**(8): p. e034665.
20. da Silva, H.S. and B.A.O. Gutierrez, *Care complexity in hospitalized elderly according to cognitive performance.* Revista Brasileira De Enfermagem, 2019. **72**: p. 134-139.
21. Bakker, M.H., et al., *Self-reported oral health problems and the ability to organize dental care of community-dwelling elderly aged >= 75years.* Bmc Oral Health, 2020. **20**(1).
22. Hoeksema, A.R., et al., *Elderly with remaining teeth report less frailty and better quality of life than edentulous elderly: a cross-sectional study.* Oral Diseases, 2017. **23**(4): p. 526-536.
23. Bakker, M.H., et al., *Are Edentulousness, Oral Health Problems and Poor Health-Related Quality of Life Associated with Malnutrition in Community-Dwelling Elderly (Aged 75 Years and Over)? A Cross-Sectional Study.* Nutrients, 2018. **10**(12).

24. Peters, L.L., et al., *Predictive validity of a frailty measure (GFI) and a case complexity measure (IM-E-SA) on healthcare costs in an elderly population.* J Psychosom Res, 2015. **79**(5): p. 404-11.
25. Yoshida, S., et al., *Correlation of patient complexity with the burden for health-related professions, and differences in the burden between the professions at a Japanese regional hospital: a prospective cohort study.* BMJ Open, 2019. **9**(2): p. e025176.
