# Supplementary material for: Evaluation of Complexity Measurement Tools for Correlations with Health-Related Outcomes, Health Care Costs and Impacts on Healthcare Providers: A Scoping Review
Source: Int J Environ Res Public Health. 2022 Dec 1;19(23):16113. doi: 10.3390/ijerph192316113 (PMC9741446; doi:10.3390/ijerph192316113)
Supplement: Supplementary file 1 [file ijerph-19-16113-s001.zip › supplementary 3.pdf]

Supplementary 3: Characteristics of the studies excluded from the qualitative syntheses

（23 Studies which were excluded in the process of RQ1）

**Study**	**Reason for exclusion**
Chen, S.S., M. Unruh, and M. Williams, Seminars in Dialysis, 2016. 29(2): p. 103-10.	Wrong population
Duane, B.G., et al., Community Dental Health, 2014. 31(4): p. 200-206.	Wrong population
Ferrer, F.M., et al., International Journal of Integrated Care (IJIC), 2016. 16(6): p. 1-2.	Not suitable publication format
Hotham, R., et al., Emergency Medicine Journal, 2022. 39(1): p. 10-15.	Could not access
Hudon, C., et al., 2021, BioMed Central. p. 1-2.	Not suitable publication format
Little, S., G. Rodgers, and J.M. Fitzpatrick, British Journal of Community Nursing, 2019. 24(2): p. 58-66.	No use of complexity measurement tool
Lobo, E. and N. Enfermería Clínica, 2010. 20(5): p. 309-312.	Not written in English
Maiden, G. and J. Nelson, International Journal of Integrated Care (IJIC), 2018. 18: p. 1-2.	Not suitable publication format
Thomas, S.J., et al., Nurse Researcher, 2016. 23(4): p. 9-13.	No use of complexity measurement tool
Thurber, S., et al., International Journal of Psychiatry in Clinical Practice, 2018. 22(1): p. 80-82.	Wrong population
Staeheli, M., et al., SAGE Open Medicine, 2017. 5: p. 2050312117712656.	Wrong population
Gottlieb, A., et al., BMC Medicine, 2013. 11: p. 194.	Wrong population

Li, Y., et al., Medical Care, 2019. 57(11): p. 875-881.	No use of complexity measurement tool
Burns, P.R. and C. Elliott, International Journal of Integrated Care (IJIC), 2016. 16(6): p. 1-3.	No clear explanation of the tool
Fink, W., G. Kamenski, and M. Konitzer, Journal of Evaluation in Clinical Practice, 2018. 24(1): p. 293-300.	No use of complexity measurement tool
Cardona, S., D.A. Escobar García, and J.A. Montoya, Rural & Remote Health, 2021. 21(4): p. 15-32.	No use of complexity measurement tool
Weiner, J.Z., et al., Journal of General Internal Medicine, 2019. 34(6): p. 818-819.	No use of complexity measurement tool
Islam, R., C. Weir, and G. Del Fiol, Methods of Information in Medicine, 2016. 55(1): p. 14-22.	No use of complexity measurement tool
Gallasch, D., et al., Physiotherapy, 2022. 114: p. 38-46.	No use of complexity measurement tool
Fung, C.H., et al., Journal of General Internal Medicine, 2008. 23(6): p. 788-93.	No use of complexity measurement tool
Swavely, D., et al., American Journal of Medical Quality, 2020. 35(2): p. 101-109.	No use of complexity measurement tool
Rosenkrantz, A.B., et al., Academic Radiology, 2018. 25(2): p. 219-225.	No use of complexity measurement tool
Grant, R.W., et al., JAMA Network Open, 2020. 3(12): p. e2029068-e2029068.	Wrong population

Appendixes(C) 2: Characteristics of the studies excluded from the qualitative syntheses

（32 Studies which were excluded in the process of RQ1）

**Study**	**Reason for exclusion**
Grant, R.W., et al., Ann Intern Med, 2011. 155(12): p. 797-804.	No use of complexity measurement tool
Grant, R.W., et al., Ann Intern Med, 2011. 155(12): p. 797-804.	Duplication
Hodgson, A., et al., Journal for Healthcare Quality, 2017. 39(2): p. 107-121.	No use of complexity measurement tool
McClane, K.S., The Journal for Advanced Nursing Practice, 2006. 20(4): p. 201-207.	No use of complexity measurement tool
Mills, P. and K. MacLure, International Journal of Integrated Care (IJIC), 2019. 19(S1): p. 1-2.	No use of complexity measurement tool
Mount, J.K., R.M. Massanari, and J. Teachman, The Journal of Collaborative Family HealthCare, 2015. 33(2): p. 137-145.	No tool development or tool reviews
Nardi, R., M. La Regina, and A. Fontanella, European Journal of Internal Medicine, 2018. 48: p. e11-e12.	No use of complexity measurement tool
O, Y. and B. G, Western Journal of Emergency Medicine: Integrating Emergency Care with Population Health, 2019. 20: p. S12-S12.	No use of complexity measurement tool
Rajkomar, A., et al., JMIR Medical Informatics, 2016. 4(4): p. e29.	No use of complexity measurement tool
Wodchis, W.P., Healthcare Quarterly, 2016. 19(2): p. 44-48.	No use of complexity measurement tool

Zhang, C., et al., International Journal of Environmental Research & Public Health, 2018. 15(4): p. 17.	No use of complexity measurement tool
Yokokawa, D., et al., BMJ Open, 2022. 12(4): p. e051891.	No tool development or tool reviews
Roberts, R.M., et al., Australian Health Review, 2007. 31(2): p. 173-183.	No use of complexity measurement tool
Lepelley, M., et al., International Journal for Quality in Health Care, 2018. 30(1): p. 32-38.	No use of complexity measurement tool
Hudon, C., et al., BMC Health Services Research, 2021. 21(1): p. 1-9.	Duplication
Oliveira, C.A., et al., PLoS ONE, 2022. 17(2): p. e0263702.	No tool development or tool reviews
Sugiyama, Y., M. Matsushima, and H. Yoshimoto, BMJ Open, 2020. 10(8): p. e034665.	No tool development or tool reviews
Marcoux, V., et al., PLoS One, 2017. 12(11): p. e0188663.	Duplication
Ghazalbash, S., et al., International Journal of Medical Informatics, 2021. 156: p. N.PAG-N.PAG.	No use of complexity measurement tool
Corrao, S., et al., Internal & Emergency Medicine, 2020. 15(4): p. 621-628.	No use of complexity measurement tool
Matzer, F., et al., PLoS ONE [Electronic Resource], 2012. 7(8): p. e41775.	No tool development or tool reviews
Davis, A.C., et al., Journal of General Internal Medicine, 2022. 37(2): p. 351-358.	No use of complexity measurement tool
Bravetti, C., et al., Annali di Igiene, 2018. 30(5): p. 410-420.	No use of complexity measurement tool

Safford, M.M., J.J. Allison, and C.I. Kiefe, J Gen Intern Med, 2007. 22 Suppl 3: p. 382-90.	No use of complexity measurement tool
Yoshida, S., et al., BMJ Open, 2017. 7(5): p. e016175.	No tool development or tool reviews
Yoshida, S., et al., BMJ Open, 2019. 9(2): p. e025176.	No tool development or tool reviews
Corrao, S., et al., Internal & Emergency Medicine, 2020. 15(4): p. 621-628.	Duplication
Arjan, K., et al., PLoS ONE, 2021. 16(3): p. e0248477.	No use of complexity measurement tool
Mutai, R., et al., BMJ Open, 2020. 10(11): p. e037282.	No tool development or tool reviews
Kamnetz, S., et al., Quality Management in Health Care, 2018. 27(4): p. 185-190.	No use of complexity measurement tool
Rosenkrantz, A.B., et al., Academic Radiology, 2018. 25(2): p. 219-225.	No use of complexity measurement tool
Pratt, R., et al., Journal of Comorbidity, 2015. 5: p. 110-119.	No tool development or tool reviews

Appendixes(C) 3: Characteristics of the studies excluded from the qualitative syntheses

（43Studies which were excluded among studies identified in the process of examining citation）

**Study**	**Reason for exclusion**
Hudon, C., et al.,BMC Health Services Research, 2021. 21(1): p. 1-9.	No outcome evaluation
Kobyłko, A., et al., Psychiatria polska, 2021. 55(6): p. 1-23.	Wrong publication format
Thurber, S., et al., International Journal of Psychiatry in Clinical Practice, 2018. 22(1): p. 80-82.	Wrong Population

Matzer, F., et al., PLoS ONE, 2012. 7(8): p. e41775.	No outcome evaluation
Liechti, F.D., et al.,BMJ Open, 2021. 11(5): p. e041205.	No outcome evaluation
Mutai, R., et al., BMJ Open, 2020. 10(11): p. e037282.	No outcome evaluation
de Jonge, P., et al., PSYCHOSOMATICS, 2001. 42(3): p. 204-212.	No use of complexity measurement tool
Gutierrez, B.A.O., H.S.D. Helena, and H.E. Shimizu, ACTA PAULISTA DE ENFERMAGEM, 2014. 27(5): p. 427-433.	No outcome evaluation
de Jonge, P., et al., Psychosomatics, 2000. 41(6): p. 505-511.	Wrong Population
Vallet, F., C. Busnel, and C. Ludwig, Recherche En Soins Infirmiers, 2019(138): p. 53-64.	Not written in English
Wild, B., et al., J Psychosom Res, 2011. 70(2): p. 169-78.	No outcome evaluation
Shukor, A.R., et al., Community Mental Health Journal, 2019. 55(8): p. 1326-1343.	Duplication
Ruiz-Miralles, M.L., et al., Anales Del Sistema Sanitario De Navarra, 2021. 44(2): p. 195-204.	Not written in English
Baird, M.A., et al., 2013, Springer New York. p. 299-324.	Wrong publication format
Boehlen, F.H., et al., Psychotherapie Psychosomatik Medizinische Psychologie, 2016. 66(5): p. 180-186.	Not written in English
Burrus, C., et al., Clinical Rehabilitation, 2021. 35(1): p. 135-144.	Wrong Population
Carpenter, L.J., et al., Australasian Psychiatry, 2021. 29(3): p. 256-260.	No outcome evaluation
Corminboeuf, Y., et al., Obesity Surgery, 2021. 31(9): p. 3996-4004.	Wrong Population

de Jonge, P., et al., Australian and New Zealand Journal of Psychiatry, 2005. 39(9): p. 795-799.	Wrong publication format
de Jonge, P. and F. Stiefel, J Psychosom Res, 2003. 54(5): p. 497-9.	Wrong Population
Duong, H.P., et al., Journal of Pain Research, 2020. 13: p. 3235-3245.	Wrong Population
Hewner, S., et al., EGEMS (Wash DC), 2017. 5(2): p. 2.	Duplication
Kishi, Y., et al., Journal of Psychosomatic Research, 2010. 69(6): p. 583-586.	Wrong Population
Kishi, Y., et al., Psychosomatics, 2004. 45(6): p. 470-476.	Wrong Population
Latour, C.H.M., et al., Nursing & Health Sciences, 2007. 9(2): p. 150-156.	Wrong publication format
Lobo, E., et al., Clinical Transplantation, 2013. 27(3): p. 417-425.	Wrong Population
Meller, W., et al., Annals of Clinical Psychiatry, 2015. 27(1): p. 39-43.	No outcome evaluation
Paoloni-Giacobino, A., et al., Journal of Pain Research, 2020. 13: p. 1289-1296.	No outcome evaluation
Scerri, M., et al., Joint Bone Spine, 2006. 73(6): p. 736-741.	Wrong Population
Smeets, R.G.M., et al., International Journal of Environmental Research and Public Health, 2021. 18(22).	No outcome evaluation
Smeets, R.G.M., et al., International Journal of Environmental Research and Public Health, 2021. 18(22).	Duplication
Stiefel, F., et al., Psychotherapy and Psychosomatics, 2008. 77(4): p. 247-256.	Wrong Population
Stiefel, F.C., et al., Medical Clinics of North America, 2006. 90(4): p. 713-+.	No outcome evaluation
van der Sluijs, J.F.V., et al., Psychosomatics, 2017. 58(4): p. 427-436.	Wrong Population
Vouilloz, A., et al., Joint Bone Spine, 2011. 78(2): p. 194-199.	Wrong Population
Huber, E., et al., Nursing Open, 2020. 7(1): p. 212-224.	No outcome evaluation

Conca, A., et al., Bmc Health Services Research, 2018. 18.	No use of complexity measurement tool
Preyde, M. and T. Chapman, Social Work in Health Care, 2007. 45(2): p. 77-95.	No use of complexity measurement tool
Huber, E., et al., Pflege, 2020. 33(3): p. 143-152.	Not written in English
Hodgins, M.J., et al., Applied Nursing Research, 2018. 41: p. 36-40.	No outcome evaluation
Chan, W.C.H., C.L.F. Chan, and M. Suen, Health & Social Work, 2013. 38(4): p. 214-221.	No use of complexity measurement tool
Carpenter, L.J., et al., Australasian Psychiatry, 2021. 29(3): p. 256-260.	No outcome evaluation
Wild, B., et al., International Journal of Geriatric Psychiatry, 2019. 34(2): p. 272-279.	No correlation evaluation
